# Extended Induction and Prognostic Indicators of Response in Patients Treated with Mirikizumab with Moderately to Severely Active Ulcerative Colitis in the LUCENT Trials

**DOI:** 10.1093/ibd/izae004

**Published:** 2024-01-25

**Authors:** Geert D’Haens, Peter D R Higgins, Laurent Peyrin-Biroulet, Bruce E Sands, Scott Lee, Richard E Moses, Isabel Redondo, Rodrigo Escobar, Theresa Hunter Gibble, Anthony Keohane, Nathan Morris, Xin Zhang, Vipin Arora, Taku Kobayashi

**Affiliations:** Department of Gastroenterology, Inflammatory Bowel Disease Centre, Amsterdam University Medical Center, Meibergdreef 9, C2-208, 1105 AZ Amsterdam, the Netherlands; Gastroenterology Clinic, Taubman Center, 1500 E Medical Center Dr, University of Michigan, Ann Arbor, MI, USA; Department of Gastroenterology, University of Lorraine, CHRU-Nancy, France; University of Lorraine, Inserm, NGERE, F-54000 Nancy, France; Division of Gastroenterology and Hepatology, McGill University Health Centre, Montreal, Quebec, Canada; Division of Gastroenterology, Icahn School of Medicine at Mount Sinai, New York, NY, USA; Digestive Health Center, University of Washington Medical Center, Seattle, WA, USA; Eli Lilly and Company, Lilly Corporate Center, Indianapolis, IN, USA; Eli Lilly Portugal, Rua Galileu Galilei 2 Lisboa 1500-392, Portugal; Lilly S.A, Avenida de la Industria, 30. 28108, Alcobendas, SpainMadrid; Eli Lilly and Company, Lilly Corporate Center, Indianapolis, IN, USA; HaaPACS GmbH, Statistics Europe, France; Eli Lilly and Company, Lilly Corporate Center, Indianapolis, IN, USA; Eli Lilly and Company, Lilly Corporate Center, Indianapolis, IN, USA; Eli Lilly and Company, Lilly Corporate Center, Indianapolis, IN, USA; Center for Advanced IBD Research and Treatment, Kitasato University Kitasato Institute Hospital, 5-9-1 Shirokane, Minato-ku, Tokyo, 108-8642, Japan

**Keywords:** mirikizumab, ulcerative colitis, IL-23 antibodies, extended induction

## Abstract

**Background:**

Efficacy and safety of mirikizumab, a p19-targeted anti-interleukin-23 monoclonal antibody, for moderately to severely active ulcerative colitis was demonstrated previously. We evaluated clinical response, baseline characteristics, and clinical status in patients not responding by 12 weeks (W) of induction who then received extended induction treatment.

**Method:**

Patients unresponsive to 300 mg of intravenous (IV) mirikizumab every 4 weeks by W12 received 3 additional 300 mg IV doses every 4 weeks. Week-4 responders received 200 mg mirikizumab every 4 weeks subcutaneously until W52. Patients responding by W12 but subsequently losing response received rescue therapy with 300 mg IV for 3 doses every 4 weeks. Logistic regression modelling was performed for patients not achieving W12 clinical response to assess baseline characteristics and W12 efficacy parameters and potential prognostic factors of clinical response at W24.

**Results:**

Of patients not achieving clinical response during induction, 53.7% achieved response following extended induction. After 52W, 72.2%, 43.1%, and 36.1% of patients achieved clinical response, endoscopic, and clinical remission, respectively. Of induction responders who subsequently lost response, 63.2% and 36.8% achieved symptomatic response and remission, respectively, after receiving rescue therapy No prior biologic or tofacitinib treatment, no immunomodulators at baseline, age older than 40 years, and W12 modified Mayo Score improvement were positively associated with a response to extended induction. The safety profile was similar to initial induction, with 38.3% treatment emergent adverse events, mostly mild.

**Conclusion:**

With “extended induction,” total of 80.3% mirikizumab-treated patients achieved clinical response by W24. Potential prognostic factors determining response include disease severity, disease phenotype, C-reactive protein, and previous biologic therapy.

## Graphical Abstract

**Graphical Abstract:**
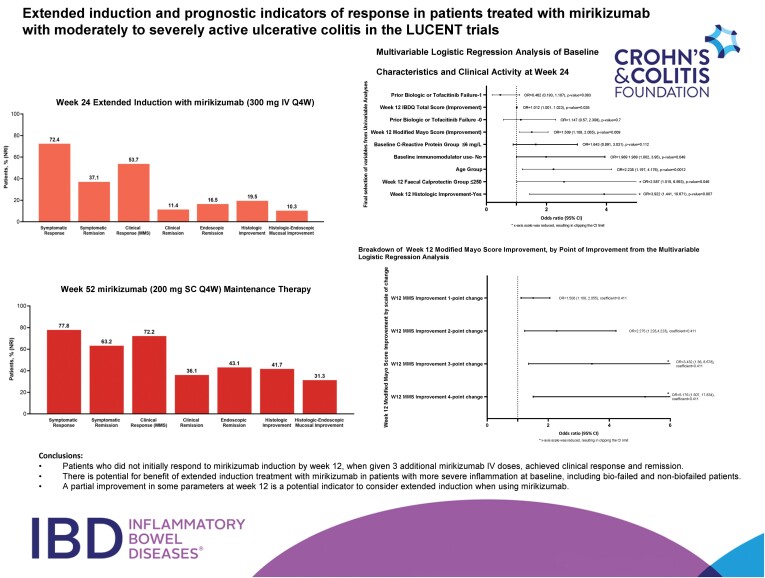

**Key Messages**

**What is already known?**

The efficacy and safety of mirikizumab for moderately to severely active UC was demonstrated in the phase 3 LUCENT trials.

**What is new here?**

For patients slower to respond to initial induction therapy, extended induction therapy with mirikizumab induced a clinical response in 53.7% of patients at W24 and clinical remission in 36.1% at W52; at W24, cumulative clinical response from both initial and extended induction was 80.3%.

**How can this study help patient care?**

Identifying a patient profile likely to respond to extended induction or reinduction treatment and maintain clinical benefit could provide a valuable option for patients with moderately to severely active UC.

## Introduction

When treating patients with ulcerative colitis (UC), response assessment determines whether to continue or switch treatment. Identifying patient characteristics or early clinical outcomes that allow prognosis of response would enable a more informed decision.

Current treatments for UC are often limited by primary nonresponse, secondary loss of response, or incomplete response.^1-3^ Primary nonresponse is defined as lack of clinical benefit during a standard induction phase, and secondary loss of response is defined as a loss of response during continued treatment (LOR).^3^ Primary nonresponse was reported in 20% to 40% of patients with IBD in clinical trials with both infliximab and adalimumab.^4,5^ Recently, percentages of primary nonresponse around 20% have been reported for tofacitinib^5^ and approximately 25% for ustekinumab.^6^ Some patients with limited or no response during the induction phase may benefit from an additional course of induction treatment before achieving clinical response.^7^ Continuing the same therapy in “delayed response” patients prevents the need to switch to another class of medication.

Mirikizumab is a human monoclonal antibody against the p19 subunit of interleukin (IL)-23, which has demonstrated achievement of clinical remission, symptomatic remission, endoscopic and histologic end points and was well tolerated following 12 weeks of induction treatment and 40 weeks of maintenance treatment in phase 3 trials of patients with moderately to severely active UC.^8-11^ In an earlier phase 2 study, extended induction treatment with mirikizumab for an additional 12 weeks resulted in clinical response in up to 50% of patients who did not have a clinical response at 12 weeks following 3 initial induction doses, and that clinical response was maintained through 52 weeks.^8^

In the phase 3 LUCENT-2 study, we explored the clinical benefit of 3 additional intravenous (IV) mirikizumab doses in patients who did not meet the response criteria following initial induction in LUCENT-1 and those who lost response while receiving maintenance therapy in LUCENT-2. Additionally, we aimed to investigate how baseline patient characteristics and clinical outcomes at week 12 impacted response at week 24, aiming to help clinicians understand what patient profile would most benefit from an extended induction treatment course on mirikizumab.

## Methods

### Patient Groups

The study design and treatment protocols of the 12-week LUCENT-1 induction study (NCT03518086) and the 40-week LUCENT-2 maintenance study (NCT03524092) have been previously described.^10,12^ In this study, the weeks of treatment were cumulative, that is, week 52 of treatment corresponds to 40 weeks of LUCENT-2 maintenance trial.

The LUCENT-1 modified intention-to-treat population (mITT) population included all randomized patients who received at least 1 dose of study treatment (excluding patients whose LUCENT-1 baseline data were collected using incorrectly transcribed patient-reported outcomes [PRO] instruments on the electronic clinical outcome assessment [eCOA]). Patients (N=1162) were randomized to placebo (N = 294) or mirikizumab (N = 868).

Of the mirikizumab-treated patients, 551 (63.5%) were clinical responders at week 12, defined as achieving ≥2-point and ≥30% decrease in the modified Mayo Score (MMS) from baseline, with rectal bleeding (RB) at 0 or 1, or ≥1-point decrease from baseline. Of these LUCENT-1 week 12 clinical responders, 544 patients were randomized to receive maintenance mirikizumab therapy or placebo in LUCENT-2.

**Figure 1.**
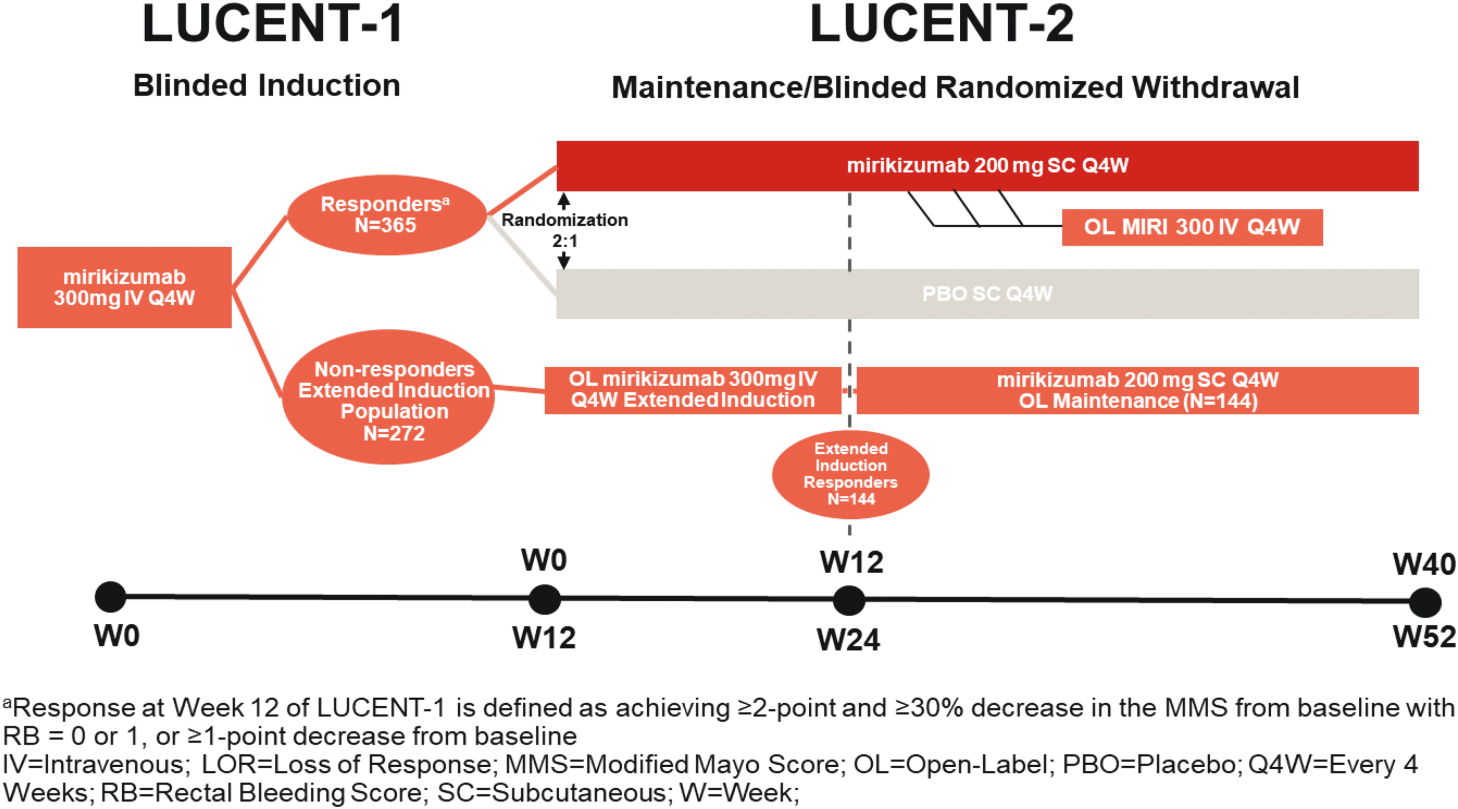
LUCENT-1 and LUCENT-2 study designs.

A total of 317 patients treated with mirikizumab in LUCENT-1 did not achieve clinical response at week 12. Of these patients, 272 completed the initial 12 weeks of induction therapy and enrolled in LUCENT-2 and received extended induction therapy (300 mg at weeks 12, 16, and 20). These patients are defined as the “extended induction population” (Figure 1). Of these patients, those who achieved clinical response at week 24 and began subcutaneous (SC) therapy in LUCENT-2 (N = 144) are defined as “extended induction responders” (Figure 1). These patients received mirikizumab maintenance therapy (open-label 200 mg of SC mirikizumab every 4 weeks [Q4W]) up to week 40 of LUCENT-2 (52 weeks of continuous treatment). Patients who did not achieve clinical response at week 24 were discontinued from the study.

Mirikizumab induction responders who experienced loss of response (LOR, N = 19) between weeks 12 and 28 of continuous treatment in LUCENT-2 could receive 3 additional doses of 300 mg of open-label IV mirikizumab Q4W. These patients are defined as the “re-induction population” (Figure 1). If no clinical benefit was achieved, patients were discontinued from the study. Loss of response was defined as ≥2-point increase from LUCENT-2 baseline in the combined stool frequency (SF) + RB scores and combined SF + RB score of ≥4 on 2 consecutive visits (≥7 days apart) and with confirmation of negative *Clostridium difficile* testing (first assessment could start as early as week 20); this was confirmed by centrally read endoscopic subscores of 2 or 3 no sooner than week 12 and no later than week 28.

The biologic-failed patient population (bio-failed) was defined as patients who had an inadequate response to, loss of response to, or were intolerant to biologic therapy or Janus kinase inhibitor (tofacitinib) for UC. Those who did not meet the definition for biologic failure were labeled as “not biologic-failed.”

### Study Oversight

All patients provided informed consent for participation in the study. The protocol, amendments, and consent documentation were approved by local ethical review boards. The study was registered at European Network of Centres for Pharmacoepidemiology and Pharmacovigilance and was conducted according to Good Pharmacoepidemiology Practices guidelines and the Declaration of Helsinki.

### Outcome Measures

Patients who underwent extended induction with mirikizumab began corticosteroid tapering if clinical response was achieved from week 24 onwards (week 12 of extended induction dosing). However, based on investigator discretion, if symptomatic improvement was evident, corticosteroid tapering could begin earlier and at any time after starting extended IV induction dosing.

The primary end points and major secondary end points for LUCENT-1 and LUCENT-2 can be found in Supplementary Table 1.

### Statistical Analyses

Baseline efficacy and safety measures were based on LUCENT-1 study baseline. Variables were analyzed in the original scale on which they were measured unless stated otherwise. Summary tables were produced to assess the percentage of patients with moderately to severely active UC achieving efficacy outcomes such as clinical response, clinical remission, symptomatic remission, histologic-endoscopic mucosal improvement (HEMI), and histologic-endoscopic mucosal remission (HEMR); (Table 1) at week 12, week 24 (week 12 of extended induction), and week 52; 40 weeks of maintenance safety analysis was performed using patients who received at least 1 dose of study treatment. Patients who discontinued treatment or were missing end point assessments were treated as nonresponders for binary efficacy end points and variables. For continuous efficacy variables, modified baseline observation carried forward (mBOCF) was applied as follows. For patients discontinuing treatment due to an adverse event (AE), the baseline observation for the end point was carried forward to the corresponding visit for all missing observations after the patient discontinued study treatment. For patients discontinuing treatment for any other reason, the last nonmissing postbaseline observation before discontinuation was carried forward to the corresponding visit for all missing observations after the patient discontinued. For all patients with sporadically missing observations prior to discontinuation, the last nonmissing observation before the sporadically missing observation was carried forward to the corresponding visit. Randomized patients without at least 1 postbaseline observation were not included for evaluation, with the exception of patients discontinuing study treatment due to an AE.

**Table 1.** Baseline demographics and disease characteristics for the mirikizumab induction responder and non-responder (extended induction) patient populations LUCENT-2 (Week 12).

	Mirikizumab Induction Responder Population	Mirikizumab Extended Induction Population^a^
	W12 N = 544	W12 N = 272
Age, mean years (SD)	42.7 (13.8)	44.0 (14.2)
Male, n (%)	318 (58.5)	182 (66.9)
Disease duration, mean years (SD)	6.8 (6.6)	7.6 (6.8)
Disease location, n (%)		
Left-sided colitis	353 (64.9)	154 (56.6)
Pancolitis	187 (34.4)	116 (42.6)
Modified Mayo Score, n (%)		
Moderate (4-6)	258 (47.4)	117 (43.0)
Severe (7-9)	286 (52.6)	154 (56.6)
Mayo endoscopic subscore: severe disease (3), n (%)	341 (62.7)	197 (72.4)
Bowel urgency severity (UNRS)		
Mean (SD)	6.1(2.13)	6.2 (2.19)
Urgency NRS ≥ 3 at baseline, n (%)	508 (93.4)	256 (94.1)
Faecal calprotectin, µg/g, median (Q1, Q3)	1565.0 (597.0, 3276.0)	1546.0 (650.0, 2912.0)
C-reactive protein (CRP), mg/L, median (Q1, Q3)	3.6 (1.3, 8.4)	5.5 (2.5, 13.6)
IBDQ total score, mean (SD)	132.4(32.80)	131.3 (33.75)
Prior UC therapy, n (%)		
Prior biologic or tofacitinib failure	192 (35.3)	147 (54.0)
Number of failed biologics or tofacitinib		
▪0	352 (64.7)	125 (46.0)
▪1	112 (20.6)	56 (20.6)
▪≥2	80 (14.7)	91 (33.4)
Prior anti-TNF failure	170 (31.3)	135 (49.6)
Prior vedolizumab failure	70 (12.9)	81 (29.8)
Prior tofacitinib failure	16 (2.9)	18 (6.6)
Baseline UC therapy, n (%)		
Corticosteroids	203 (37.3)	118 (43.4)
Immunomodulators	117 (21.5)	77 (28.3)
Aminosalicylates	412 (75.7)	202 (74.3)

Abbreviations: W, week; BMI, body mass index; IBDQ, Inflammatory Bowel Disease Questionnaire; IV, intravenous; N, number patients; Q, quartile; SC, subcutaneous; SD, standard deviation; TNF, Tumor Necrosis Factor; UC, ulcerative colitis; UNRS, Urgency Numeric Rating Scale.

^a^Mirikizumab induction nonresponders who received another 3 IV mirikizumab doses 4QW.

### Regression Modelling: Identifying Variables that Impact Response

Logistic regression modelling was performed with clinical response at week 24 as the outcome among the population of patients that did not achieve clinical response after 12 weeks of mirikizumab induction therapy. Categories of prognostic indicators considered for the model included baseline patient and disease characteristics (eg, age, weight, sex, prior therapy, disease activity) and efficacy parameters from the week-12 visit. These consisted of both continuous and categorical variables.

For each individual prognostic variable, a univariable logistic regression analysis was performed to assess which variables were associated with week-24 clinical response.

The number of potential prognostic indicators was large, and many of them were correlated with each other. To develop a small parsimonious multivariable model, a forward and backward stepwise regression method was used to select a model that minimizes the Akaike Information Criterion.^13,14^ The Firth correction^15^ was used for all logistic regression models.

### Pharmacokinetic Analysis

The geometric mean and geometric coefficient of variance (CV%) for trough concentration was calculated using the observed mirikizumab serum concentration data at week 12, week 24, week 52, and before loss of response for the responder and nonresponder groups. Trough level was defined as 21 to 35 days from last dose.

## Results

### Baseline Demographics and Disease Characteristics for the Mirikizumab Induction and Extended Induction Patient Population

The demographics and disease burden in the extended induction population at week 12 showed a higher disease burden and a history of prior failure to multiple biologics or tofacitinib in the induction responders.

Compared with induction responders, a numerically higher percentage of the 272 patients who did not achieve a clinical response at week 12 of LUCENT-1 had a baseline Mayo endoscopic subscore of 3, had a history of biologics or tofacitinib failure, were using corticosteroids, and had pancolitis (Table 1). Also, the mean (standard deviation) disease duration at baseline tended to be numerically greater in the extended induction population compared with induction responders (Table 1).

### Efficacy Clinical Outcomes for the Mirikizumab Extended Induction Patient Population

#### Week 12

**Figure 2.**
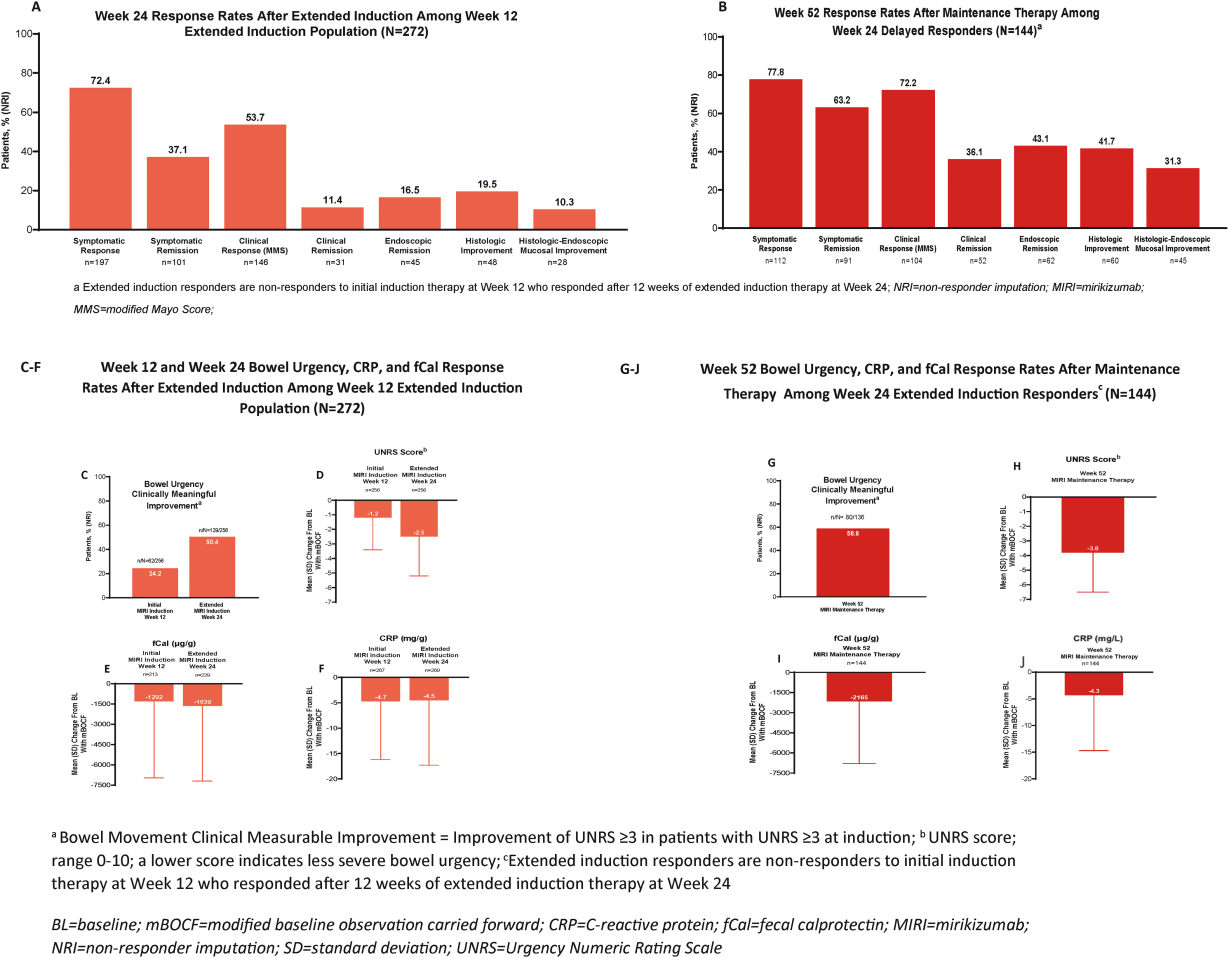
Week 24 (A) and week 52 (B) response rates among week 12 extended induction population (N = 272); week 12 and week 24 bowel urgency (C-D), CRP (E), and fCal (F) response rates after extended induction among week 12 extended induction population (N = 272), and week 52 bowel urgency (G-J), CRP (G), and fCal (H) response rates after maintenance therapy among week 24 extended induction responders (N = 144).

Patients not achieving protocol-defined criteria for response at week 12 may still show some degree of improvement at week 12. Week-12 response rates are seen in Figure 2C-F and Table 2. Symptomatic response, symptomatic remission, endoscopic remission, histologic improvement, histologic-endoscopic improvement, clinically meaningful improvement in bowel urgency, and a decrease in the bowel urgency severity scores (UNRS^16^: an 11-point patient-reported scale ranging from 0 [no urgency] to 10 [worst possible urgency] that is used to measure bowel urgency in the past 24 hours, Supplementary Table 1) were observed among the Extended Induction Population (N = 272).

**Table 2.** Clinical outcomes and trough levels at weeks 12, 24 and 52 for patients receiving mirikizumab extended induction.

Outcomes for Mirikizumab	Mirikizumab Extended Induction Population 300 mg Mirikizumab IV, Q4W N = 272^a^		Mirikizumab Extended Induction Responders 200 mg Mirikizumab SC, Q4W N = 144^b^
	W12 (of continuous mirikizumab treatment)	W24 (of continuous mirikizumab treatment)	W52 (of continuous mirikizumab treatment)
Clinical remission (NRI response) *n*, (%)	0	31 (11.4)	52 (36.1)
Clinical response (MMS; NRI response) *n*, (%)	0	146 (53.7)	104 (72.2)
Symptomatic response (NRI response) *n*, (%)	83 (30.5)	197 (72.4)	112 (77.8)
Symptomatic remission (NRI response) *n*, (%)	13 (4.8)	101 (37.1)	91 (63.2)
HEMI (NRI response) *n*, (%)	13 (4.8)	28 (10.3)	45 (31.3)
Histologic improvement (NRI response) *n*, (%)	40 (14.7)	53 (19.5)	60 (41.7)
Endoscopic remission (NRI response) *n*, (%)	21 (7.7)	45 (16.5)	62 (43.1)
fCal change from baseline, (mBOCF; mean [SD])	-1291.9 (5664.2)	-1638.8 (5558.9)	-2164.8 (4631.3)
CRP change from baseline, (mBOCF; mean [SD])	-4.7 (11.5)	-4.5 (12.8)	-4.3 (10.4)
BU Clinically meaningful Improvement (NRI response) n/Nx, (%)	62/256 (24.2)	129/256 (50.4)	80/136 (58.8)
BU change from baseline (mBOCF; mean [SD])	-1.2 (2.2)	-2.5 (2.7)	-3.8 (2.7)
Trough levels Geometric Mean (Geometric CV%) concentration (ug/mL)			
All patients	2.16 (142)^c^	2.40 (131)^d^	1.73(106)^e^
Responders	NA	2.70 (134)	1.77 (112)
Nonresponders	NA	2.06 (124)	1.60(89)

Abbreviations: BU, bowel urgency; CRP, C-reactive protein; fCal, fecal calprotectin; HEMI, histologic-endoscopic mucosal improvement; IV, intravenous; mBOCF, modified baseline observation carried forward; MMS, modified Mayo-Score; N, patient population; *n*, patient subpopulation; Nx, number of patients with Urgency Numeric Rating Scale ≥3 at induction baseline; NRI, nonresponse imputation; Q4W, every four weeks; SC, subcutaneous; W, weeks.

^a^Patients could continue on mirikizumab maintenance up to W52 if they had achieved clinical response at W24.

^b^Two of 146 responders did not enter open-label maintenance study.

^c^Patient N = 247.

^d^Patient N = 195.

^e^Patient N = 99.

At the end of the induction period in LUCENT-1, patients who achieved clinical response, and were randomly assigned to either mirikizumab 200 mg SC Q4W or placebo, had a geometric mean (geometric CV%) trough concentration of 3.72 (107%) µg/mL (Table 2). Patients who did not achieve clinical response and received extended induction treatment of 300 mg IV of mirikizumab at Q4W had a geometric mean (geometric CV%) trough concentration of 2.16 (142%) µg/mL at the end of the induction period in LUCENT-1 (Table 2).

#### Week 24

At week 24, patients who received mirikizumab extended induction treatment (300 mg IV Q4W) showed improvement and exhibited clinical response, clinical remission, symptomatic response, symptomatic remission, histologic improvement, endoscopic remission, and clinically meaningful bowel urgency improvement (Figures 2A-C, 2BTable 2). At week 24, 68/147 (46.3%) of the bio-failed patients and 78/125 (62.4%) of the not bio-failed patients achieved clinical response, whereas 12 (8.2%) patients and 19 (15.2%) achieved clinical remission, respectively (Supplementary Table 2).

Overall, patients who received mirikizumab extended induction treatment had geometric mean (geometric CV%) trough concentration of 2.40 (131%) µg/mL (Table 2). The serum concentration of mirikizumab at week 24 is slightly higher in responders compared with nonresponders of the extended induction treatment at week 24 (geometric mean (geometric CV%) trough concentration of 2.7 (134%) µg/mL) and the nonresponders to extended induction (geometric mean [geometric CV%] trough concentration of 2.06 [127%] µg/mL).

#### Week 52

The extended induction responders at week 24 who entered open-label maintenance (200 mg mirikizumab SC, Q4W) experienced continuing improvement across all outcomes. At week 52, more patients achieved clinical remission, clinical response, symptomatic remission, and symptomatic response and had endoscopic and histologic improvement (Figure 2B, Table 2). More patients had a clinically meaningful improvement in bowel urgency with an additional decrease in bowel urgency severity (Figure 2G, H, Table 2).

At week 52, 43 (29.9%) extended induction responder patients achieved corticosteroid-free remission (defined as clinical remission at week 52, achieving symptomatic remission by week 40, and with no corticosteroid use for ≥12 weeks prior to week 52). In addition, at week 52, among extended induction responder patients, 51 (35.4%) achieved clinical remission and were off corticosteroids for at least 90 days prior (extended induction corticosteroid-free clinical remission defined as corticosteroid-free remission at LUCENT-2 week 52 for at least 90 days among extended induction responder patients who achieved clinical remission).

The data for the bio-failed and not bio-failed subgroups were similar in the week 52 corticosteroid-free remission group, as well as the extended induction corticosteroid-free clinical remission group at week 52 (Supplementary Table 3).

There were 52 extended induction responder patients who achieved clinical remission at week 52, of which, 51 (98.1%) were off corticosteroids for at least 90 days prior to the week 52 end point.

At week 52, 46 (69.7%) bio-failed patients and 58 (74.4%) not bio-failed patients receiving extended induction treatment maintained clinical response, whereas 23 of 66 (34.8%) patients and 29 of 78 (37.2%) achieved clinical remission, respectively (Supplementary Table 2).

Overall, for the extended induction responders at week 24, the geometric mean trough levels (geometric CV%) at week 52 (N = 120) were 1.74 ug/mL (101%; Table 2).

### Extended Induction Patient Population: Baseline and Week 12 Outcomes Associated With Week 24 Clinical Response

**Figure 3.**
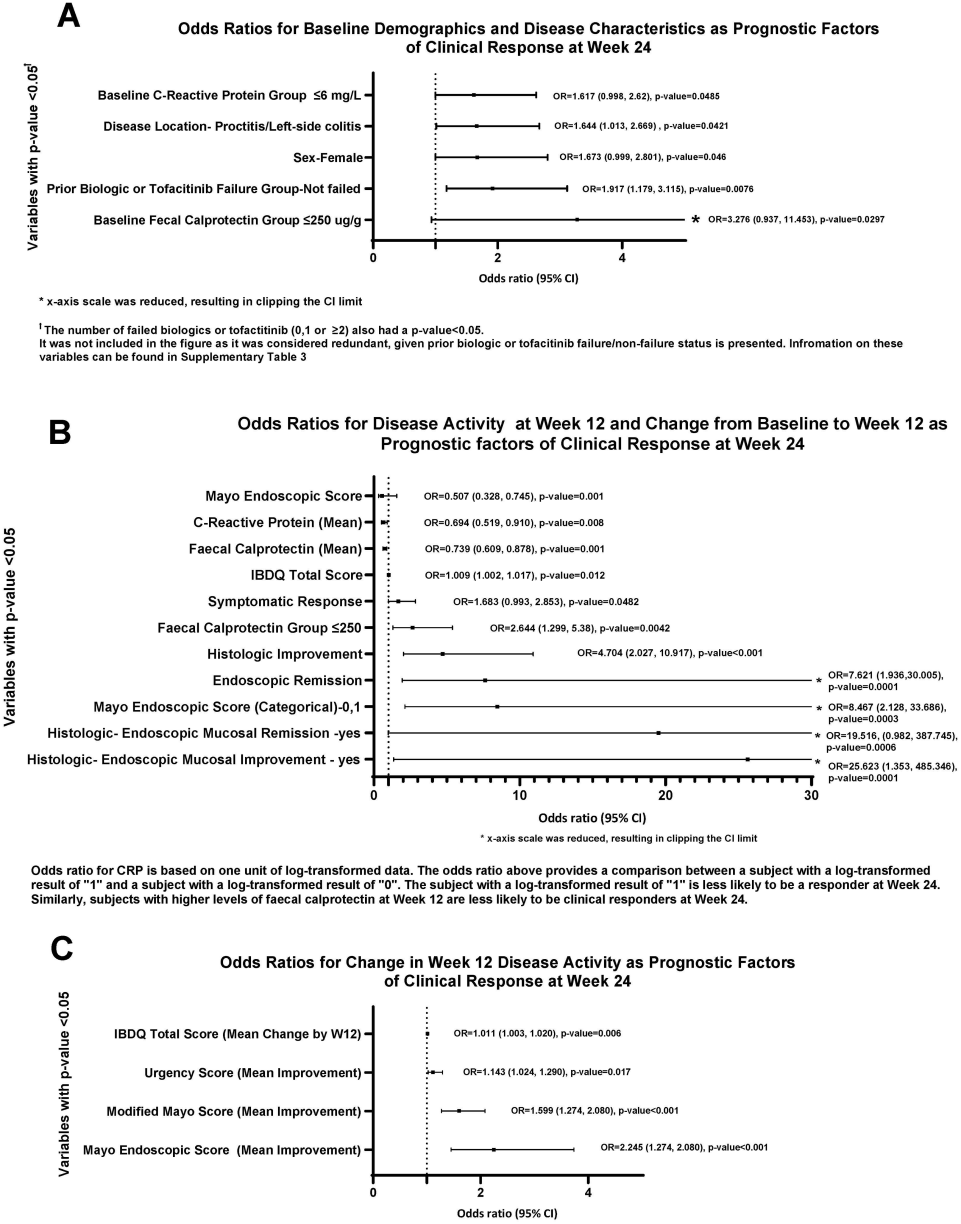
A-C, Odds ratios using week 12 baseline demographics and disease characteristics (A), disease activity, disease activity and change from baseline (B), and odds ratios for change in week 12 disease activity (C) as prognostic factors of clinical response at week 24.

The univariable logistic regression analysis using disease demographics and baseline disease characteristics from the LUCENT-1 study identified the following prognostic factors favoring clinical response at week 24 (ie, a response to extended induction): female gender (increased odds of responding of 1.7; 95% confidence interval [CI], 1.0-2.8; Figure 3A), disease location of proctitis/left-sided colitis (increased odds 1.6; 95% CI, 1.0-2.7) compared with patients with pancolitis, biologic and tofacitinib not-failure (increased odds 1.9; 95% CI, 1.2-3.1; Figure 3A, Supplementary Table 4), and the number of prior bio-failed treatments (0, 1, or ≥2). Indeed, the odds ratio (OR) for achieving response after extended induction was 1.9 (95% CI, 1.2-3.1) for not bio-failed patients and 0.7 (95% CI, 0.37,1.4) for those who have failed 1 targeted therapy (Supplementary Table 4). Both a baseline fecal calprotectin (fCal) level ≤250 ug/g (OR, 3.3; 95% CI, 0.94-11.5) and a baseline C-reactive protein (CRP) level ≤6 mg/L (OR 1.6; 95% CI, 1.0-2.6) were associated with increased odds of achieving clinical response (Figure 3A). The univariable associations between each potential prognostic factor and clinical response can be seen in Supplementary Table 4.

### Univariable Analysis Using Disease Activity at Week 12 and Change From Baseline to Week 12 as Prognostic Factors of Clinical Response at Week 24

The univariable regression analysis using disease activity at week 12 and change from baseline to week 12 of LUCENT-1 study to identify prognostic factors of clinical response at week 24 (ie, response to extended induction) indicated that lower Mayo Endoscopic Subscore (MES), categories of MES, and higher Inflammatory Bowel Disease Questionnaire (IBDQ) total score, bowel urgency remission, fCal, fCal threshold level (250 ug/g), CRP, CRP threshold level (6mg/L), endoscopic remission, histologic improvement and symptomatic response, IBDQ total score change from baseline, UNRS improvement, MMS improvement, and MES improvement were all variables with a statistically significant (*P* < .05) association with clinical response at week 24 (ie, a response to extended induction, Figure 3B).

Achieving endoscopic remission (MES of 0 or 1) at week 12 (OR, 7.6; 95% CI, 1.9-30.0), histologic improvement (OR, 4.7; 95% CI, 2.0-10.9), fCal ≤250 ug/g (OR, 2.6; 95% CI, 1.3-5.4) at week 12, and lower CRP levels at week 12 (OR, 0.7; 95% CI, 0.5-0.9; Supplementary Table 5; Figure 3B) were associated with the increased odds of achieving clinical response at week 24.

Having achieved bowel urgency improvement and symptomatic responses at week 12 were also associated with an increased odds (1.1, 95% CI, 1.0-1.3; and 1.7, 95% CI, 1.0-2.9, respectively) of clinical response at week 24 (Figure 3B, C). The univariable associations between each potential prognostic factor and clinical response are shown in Supplementary Table 5.

The univariable regression analysis using change from baseline to week 12 of LUCENT-1 study to identify prognostic factors of clinical response at week 24 showed that for Mayo Endoscopic Subscore improvement, MMS, UNRS, and IBDQ total score change, the higher the improvement, the higher the odds of achieving clinical response. The progressions of improvement are seen in Figure 3C for each of these factors.

All variables from the univariable analyses based on baseline demographics and LUCENT-1 week-12 disease activity were considered for the multivariable analysis. Among the Extended Induction Population, all patients achieving HEMI/HEMR (N = 13 and N = 10 respectively) at week 12 also achieved clinical response at week 24. As a result, these variables could not be included in the multivariable modelling steps for computational reasons. The MMS categorical variable was not considered for the final model because it is too strongly correlated with the endoscopic remission variable. C-reactive protein and fCal continuous variables were omitted in favor of corresponding categorical variables. The week-12 Mayo endoscopic categorical variable was omitted, as corresponding continuous variable was included.

Consequently, there were 8 variables selected for the multivariable logistic regression model by using a stepwise selection algorithm (Supplementary Table 7). Continuous variables in the model were MMS improvement (baseline to week 12) and IBDQ total score improvement (baseline to week 12). Binary variables were histologic improvement at week 12 (yes/no), age group (<40 or ≥40), number of previous failed advanced treatments (0, 1, or ≥2), baseline immunomodulator use (yes/no), fCal group at week 12 (≤250 ug/g, >250 ug/g), and levels of baseline CRP (≤6 mg/L, >6 mg/L).

**Figure 4.**
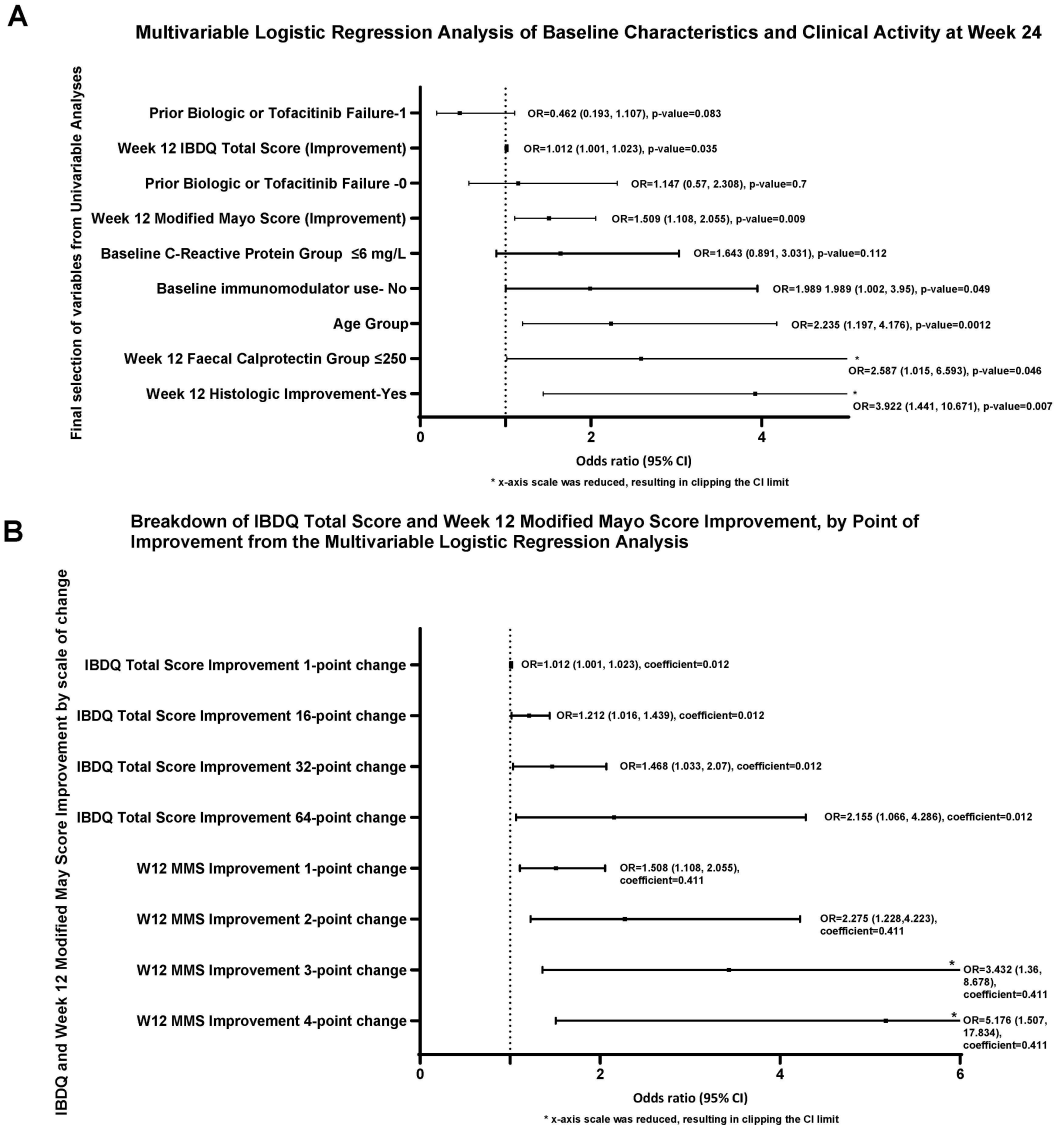
A-B, Odds ratio of baseline characteristics and clinical activity at week 24 (A) and breakdown of IBDQ total score and week 12 modified Mayo score improvement (B), by point of improvement from the multivariable logistic regression analysis.

Based on this model, age 40 years and older was associated with increased odds (2.2; 95% CI, 1.2-4.2) of achieving clinical response at week 24, followed by patients without concomitant used of immunomodulators at baseline (OR, 2.0; 95% CI, 1.0-4.0). Having baseline CRP levels ≤6 mg/L and no prior experience of biologic or tofacitinib failure were also associated with increased odds (1.6, 95% CI, 0.9-3.0; and 1.1, 95% CI, 0.6, 2.3, respectively) of achieving clinical response by week 24 (Figure 4A).

When evaluating week-12 change from baseline disease activity, achieving histologic improvement by week 12 had increased odds (3.9; 95% CI, 1.4-10.7) of achieving clinical response at week 24, followed by having achieved fCal level ≤250 ug/g by week 12 (OR, 2.6; 95% CI, 1.0-6.6; Figure 4A).

A 1-point improvement from baseline to week 12 in the MMS was associated with increased odds (1.5; 95% CI, 1.1-2.1) of achieving clinical response at week 24. Similarly, a 2-, 3-, and 4- point improvement in MMS corresponded respectively to a 2.3 (95% CI, 1.2-4.2), 3.4 (95% CI, 1.4-8.7), and 5.2 (95% CI, 1.5-17.8) increase in the odds of achieving clinical response. Patients with a 16-point improvement from baseline to week 12 in the IBDQ total score were associated with increased odds (1.2; 95% CI, 1.0-1.4) of achieving clinical response. Similarly, a 32- and 64-point improvement in IBDQ total score corresponded respectively to a 1.5 (95% CI, 1.0-2.1) and 2.2 (95% CI, 1.1-4.3) odds ratios (Figure 4B).

### Clinical Outcomes for the Mirikizumab Loss of Response Maintenance Patient Population

Among the 365 mirikizumab week-12 induction responders randomly assigned to mirikizumab maintenance treatment, 19 (5.2%) patients experienced a loss of response during maintenance and received 3 re-induction doses of rescue therapy with 300 mg of open-label mirikizumab IV. Of the rescued patients, 12 (63.2%) patients regained symptomatic response, and 7 (36.8%) achieved symptomatic remission after 12 weeks of rescue therapy.

Patients who did not lose response and remained on 200 mg SC Q4W throughout LUCENT-2 had a geometric mean (geometric CV%) trough concentration of 2.21 (101%) µg/mL at week 52. Mirikizumab trough levels in patients who were receiving 200 mg SC Q4W but lost response and were reinduced with 300 mg IV Q4W for 3 doses had a geometric mean (geometric CV%) trough concentration up to the point of losing response of 1.83 (121%) µg/mL (prior to reinduction). Due to the small sample size (N = 10 patients), no interpretation can be made in regard to the trough level impact in the small LOR population.

### Safety

Reported treatment emergent adverse events (TEAEs) for extended induction patients were low in number and frequency (38.3). At week 24, most TEAEs were mild (21.4%), while only 5.4% were serious. There were 3.2% of patients who discontinued due to TEAEs, with 1 patient having elevations of the alanine aminotransferase and total bilirubin levels that met the criteria for Hy’s law. No other cause to explain the hepatic laboratory abnormalities was identified. Hepatic enzyme and bilirubin levels returned to normal after the patient discontinued mirikizumab. No new safety signals or deaths were reported (Supplementary Table 8).

## Discussion

Some patients with active UC respond slower to induction treatment and may benefit from an extended course of induction treatment to achieve clinical response or remission. Additionally, some patients experience secondary LOR (disease flare), resulting in efforts to optimize existing treatment or switch to another therapeutic class.

The efficacy and safety of mirikizumab for moderately to severely active UC were already demonstrated in the LUCENT trials.^10^ After 12 weeks of induction with 300 mg of mirikizumab IV, 63.5% of patients achieved clinical response.

Our results from the LUCENT trials demonstrated that an extended induction strategy with mirikizumab induced response in about half of the patients, so at the end only approximately 19.7% were true primary nonresponders to mirikizumab. In this study, adding the benefit of “extended induction,” a total of 697 of 868 (80.3%) mirikizumab-treated patients achieved clinical response by week 24.

This current LUCENT study confirms the phase 2 study findings showing that extended doses of mirikizumab for an additional 12 weeks produced a clinical response in up to 50% of patients who did not have a clinical response to 12 weeks of induction doses and that most of the responders to the extended doses maintained clinical response for up to 52 weeks.^8^

Identifying patients likely to respond to treatment is an important unmet need for health care providers to optimally manage their patients with UC.^10^ Exploring clinical variables as prognostic factors, female sex (OR, 1.7; 95% CI, 0.999-2.801), lower disease activity “fecal calprotectin ≤250ug/g” (OR, 3.3; 95% CI, 0.937, 11.453]) “baseline CRP level ≤6 mg/L” (OR, 1.6; 95% CI, 0.998-2.62), as well as the variables left-sided colitis (OR, 1.6; 95% CI, 1.013-2.669) and no prior failed exposure to biologics or tofacitinib (OR, 1.9; 95% CI, 1.179-3.115) were associated in the univariable analysis with a higher likelihood of clinical response at week 24 following extended induction (Figure 3A). Moreover, a decrease in Partial Mayo Score at the end of induction (suggesting onset of clinical benefit) was associated with achieving clinical response during extended induction.^17^ Other outcome measures of clinical benefit at week 12 (endoscopic remission, histologic improvement, fCal levels ≤250 ug/g, bowel urgency remission, symptomatic response, and improvement from MMS or IBDQ baseline of at least 1 point) were also associated with clinical response at week 24 (Figure 3A, 3B, Supplementary Table 5).

Patients who achieved HEMI or HEMR by week 12 without clinical response all achieved clinical response by week 24, suggesting clinical response may be delayed but follows histologic and endoscopic end points.

In a stepwise selection algorithm for the multivariable logistic regression model, we identified “no prior biologic or tofacitinib failure,” no immunomodulators at baseline, and age older than 40 years as baseline demographics and week 12 MMS improvement (baseline to week 12) to be positively associated with clinical response at week 24.

In summary, we identified that, as in previous papers, a few factors may have a potential role in the prediction of the response, including disease behavior/phenotype, disease severity, C-reactive protein, and previous biologic therapy.^18^ But there is not one single marker fulfilling all criteria for being an appropriate prognostic indicator of response.

Clinical trial criteria for response were very stringent in the LUCENT trials. At week 12, some patients who were assessed as clinical nonresponders did demonstrate some clinical benefit with treatment, including improvement of rectal bleeding, stool frequency, and bowel urgency. Some of these patients assessed as nonresponders had achieved endoscopic remission and histologic improvement. Adhering to the strict clinical trial criteria of not reaching the clinical response end point despite demonstrating some clinical benefit would have resulted in discontinuation from the study. Based on our findings, if improvement in these identified parameters is observed, extended induction should be considered. Evidence of therapeutic response as assessed by change in symptoms and/or objective signs (such as fCal and/or CRP) can help guide a physician’s decision when to continue treatment with mirikizumab through an extended induction period.

The mean trough concentrations in patients who were responders after extended induction at week 24 or week 52 are slightly higher than the nonresponders (Table 2). However, the small differences are not considered clinically meaningful given the large variability (CV range from 89%-142%). In addition, data have shown that within the exposure range tested in the LUCENT trials, mirikizumab efficacy response is not sensitive to drug exposure, Therefore, the different response status after the extended induction is unlikely due to mirikizumab drug exposure.

Optimization via dose escalation is a common treatment approach with advanced therapies for UC. The most reported reasons (79%) for dose escalation are partial response, no response, or loss of response.^19^ The strategy in the LUCENT trials for recapture of response of LOR patients during SC maintenance was to administer 3 doses of 300 mg of mirikizumab IV Q4W. However, discontinuation rates during maintenance treatment (6.0%) were low, and hence there was a limited number of patients receiving open-label rescue. However, the benefit of recapturing response of LOR patients and preventing a need for a switch in medication strategy was evident, as was shown for the more than 60% that regained symptomatic response, having the possibility to resume SC maintenance.

Adverse events observed during extended induction, subsequent maintenance, and LOR rescue period were consistent with the mirikizumab ulcerative colitis safety profile to date and similar in type and severity to those reported previously.^10^ One patient had hepatic enzyme elevations consistent with Hy’s law during this period and fully recovered with no sequelae after discontinuation of mirikizumab. No significant differences in safety were observed between responders at week 12 and responders at week 24 of continuous treatment.

The strengths of the LUCENT-1 and LUCENT-2 studies are their randomized, double-blind design with sufficient patient numbers and power to test the primary and major secondary objectives.^10^ Potential limitations of the study for the extended induction and loss of response populations are the open-label nature of the extended induction, subsequent maintenance periods, and the absence of a placebo comparator arm in this subpopulation. Also, due to the persistence in treatment of the majority of the LUCENT-2 population, there were low numbers of patients who were reinduced with mirikizumab; however, these caveats may more closely reflect real-world clinical practice.^20^

Univariable and multivariable analyses conducted in the present study are based on an open label cohort with no control group. These analyses have attempted to provide useful associations between baseline demographics and baseline disease characteristics, as well as early disease activity and improvement to help guide clinicians to an optimal treatment decision and choice for the patient. However, without a control group, we have not demonstrated definitively that the reason the patients improved was due to the use of extended induction therapy. As such, we have chosen to describe the analysis as prognostic instead of predictive.

## Conclusions

In conclusion, the results suggest that some patients with active UC respond slower to induction treatment and may benefit from an extended course of induction treatment with mirikizumab to achieve clinical response or remission. Adding the benefit of extended induction, a total of 80.3% mirikizumab-treated patients achieved clinical response by week 24. In addition, optimization of existing treatment with mirikizumab reinduction, for the small number of patients experiencing a secondary LOR, may mitigate the need to switch to a different therapeutic class. Factors that may have a potential prognostic role in the determination of response include disease severity, disease phenotype, C-reactive protein, and previous biologic therapy. This information could be helpful in clinical management decisions for extended and reinduction in patients with moderately to severely active UC.

## Supplementary Data

Supplementary data is available at *Inflammatory Bowel Diseases* online.

izae004_suppl_Supplementary_Tables

## Data Availability

Lilly provides access to all individual participant data collected during the trial, after anonymization, with the exception of pharmacokinetic or genetic data. Data are available by request 6 months after the indication studied has been approved in the US and EU and after primary publication acceptance, whichever is later. No expiration date of data requests is currently set once data are made available. Access is provided after a proposal has been approved by an independent review committee identified for this purpose and after receipt of a signed data sharing agreement. Data and documents, including the study protocol, statistical analysis plan, clinical study report, and blank or annotated case report forms will be provided in a secure data sharing environment. For details on submitting a request, see the instructions provided at www.vivli.org.

## References

[1] Sands BE , Chen J, Penney M, et al 830 Initial evaluation of MEDI2070 (specific anti-IL-23 antibody) in patients with active Crohn’s disease who have failed anti-TNF antibody therapy: a randomized, double-blind placebo-controlled phase 2A induction study. Gastroenterology. 2015;148(4):S-163.

[2] Fine S , Papamichael K, Cheifetz AS. Etiology and management of lack or loss of response to anti-tumor necrosis factor therapy in patients with inflammatory bowel disease. Gastroenterol Hepatol. 2019;15(12):656-665. PMC6935028 31892912

[3] Roda G , Jharap B, Neeraj N, Colombel J-F. Loss of response to anti-TNFs: definition, epidemiology, and management. Clin Transl Gastroenterol. 2016;7(1):e135. 26741065 10.1038/ctg.2015.63 PMC4737871

[4] Ben-Horin S , Chowers Y. Tailoring anti-TNF therapy in IBD: drug levels and disease activity. Nat Rev Gastroenterol Hepatol. 2014;11(4):243-255. 24393836 10.1038/nrgastro.2013.253

[5] Attauabi M , Dahl EK, Burisch J, et al Comparative onset of effect of biologics and small molecules in moderate-to-severe ulcerative colitis: a systematic review and network meta-analysis. eClinicalMedicine. 2023;57(1):101866. 36864986 10.1016/j.eclinm.2023.101866 PMC9971510

[6] Lucaciu LA , Constantine-Cooke N, Plevris N, et al Real-world experience with tofacitinib in ulcerative colitis: a systematic review and meta-analysis. Ther Adv Gastroenterol. 2021;14:1-16. 10.1177/17562848211064004 PMC8721385 34987608

[7] Honap S , Al-Hillawi L, Baillie S, et al Ustekinumab for the treatment of moderate to severe ulcerative colitis: a multicentre UK cohort study. Frontline Gastroenterol. 2022;13(6):517-523. 36250172 10.1136/flgastro-2022-102168 PMC9555129

[8] Sandborn WJ , Ferrante M, Bhandari BR, et al Efficacy and safety of continued treatment with mirikizumab in a phase 2 trial of patients with ulcerative colitis. Clin Gastroenterol Hepatol. 2022;20(1):105-115.e14. 32950748 10.1016/j.cgh.2020.09.028

[9] Sandborn WJ , Ferrante M, Bhandari BR, et al Efficacy and safety of mirikizumab in a randomized phase 2 study of patients with ulcerative colitis. Gastroenterology. 2020;158(3):537-549.e10. 31493397 10.1053/j.gastro.2019.08.043

[10] D’Haens G , Dubinsky M, Kobayashi T, et al; LUCENT Study Group. Mirikizumab as induction and maintenance therapy for ulcerative colitis. N Engl J Med. 2023;388(26):2444-2455. 37379135 10.1056/NEJMoa2207940

[11] D’Haens G , Kobayashi T, Morris N, et al OP26 efficacy and safety of mirikizumab as induction therapy in patients with moderately to severely active ulcerative colitis: results from the phase 3 LUCENT-1 study. J Crohns Colitis. 2022;16(Supplement_1):i028-i029. PMC9122067 35610995

[12] Magro F , Pai RK, Kobayashi T, et al Resolving histological Inflammation in ulcerative colitis with mirikizumab in the LUCENT induction and maintenance trial programmes. J Crohns Colitis. 2023;17(9): 1457-1470. 37057827 10.1093/ecco-jcc/jjad050 PMC10588772

[13] R Core Team (2022). *R:* A language and environment for statistical computing. R Foundation for Statistical Computing, Vienna, Austria. https://www.R-project.org/.

[14] Akaike H. A new look at the statistical model identification. IEEE Trans Autom Control. 1974;19(6):716-723.

[15] Firth D. Bias reduction of maximum likelihood estimates. Biometrika. 1993;80(1):27-38.

[16] Dubinsky MC , Irving PM, Panaccione R, et al Incorporating patient experience into drug development for ulcerative colitis: development of the Urgency Numeric Rating Scale, a patient-reported outcome measure to assess bowel urgency in adults. J Patient Rep Outcomes. 2022;6(1):31. 35362902 10.1186/s41687-022-00439-w PMC8975984

[17] Dubinsky MC , Magro F, Steinwurz F, et al Association of C-reactive protein and partial Mayo Score with response to tofacitinib induction therapy: results from the Ulcerative Colitis Clinical Program. Inflamm Bowel Dis. 2023;29(1):51-61. 35380664 10.1093/ibd/izac061 PMC9825285

[18] Gisbert JP , Chaparro M. Predictors of primary response to biologic treatment (anti-TNF, vedolizumab, and ustekinumab) in patients with inflammatory bowel disease: From basic Science to clinical practice. J Crohns Colitis. 2020;14(5):694-709. 31777929 10.1093/ecco-jcc/jjz195

[19] Annese V , Nathwani R, Alkhatry M, et al Optimizing biologic therapy in inflammatory bowel disease: a Delphi consensus in the United Arab Emirates. Therap Adv Gastroenterol. 2021;14:17562848211065329. 10.1177/17562848211065329 PMC8721421 34987611

[20] European Medicines Agency. EMA Website. Accessed June 23, 2023. https://www.ema.europa.eu/en/medicines/human/EPAR/omvoh

