# Supplementary material for: Extended Induction and Prognostic Indicators of Response in Patients Treated with Mirikizumab with Moderately to Severely Active Ulcerative Colitis in the LUCENT Trials
Source: Inflamm Bowel Dis. 2024 Jan 25;30(12):2335–46. doi: 10.1093/ibd/izae004 (PMC11630349; doi:10.1093/ibd/izae004)
Supplement: izae004_suppl_Supplementary_Tables [file izae004_suppl_supplementary_tables.docx]

## Supplementary Materials

**Supplementary Table1: Clinical Outcome Definitions**

| **Outcome** | **Definition** |
| --- | --- |
| Clinical Response | ≥2-point and ≥30% decrease in MMS from baseline; RB=0 or 1 or, RB ≥1-point decrease from baseline |
| Clinical Remission | SF=0 or SF=1 with ≥1-point decrease in MMS from baseline; RB=0; and ES=0 or 1 (excluding friability) |
| Symptomatic Response | Defined as at least a 30% decrease from baseline in the composite clinical endpoint of the sum of stool frequency and rectal bleeding subscores |
| Symptomatic Remission | SF=0 or SF=1 with ≥1-point decrease in MMS from baseline; RB=0 |
| Corticosteroid-free Remission | Clinical remission at LUCENT-3 W52, achieving symptomatic remission by week 40, and with no corticosteroid use for ≥12 weeks prior to W52 |
| Extended Induction Corticosteroid-free Remission | Corticosteroid- free remission at LUCENT-3 W52 for at least 90 days among extended induction responder patients who achieved clinical remission |
| Endoscopic Remission | ES=0 or 1 (excluding friability); score ranges 0 to 3 a lower score indicates less mucosal damage |
| Histologic Improvement | Geboes histological subscores of:   - 0 (None) or 1 (<5% of crypts involved) for parameter 3 (neutrophils in epithelium), and - 0 (None) for parameter 4 (crypt destruction), and - 0 (None) for parameter 5 (erosion or ulceration) |
| Histologic- Endoscopic Mucosal Improvement (HEMI) | Geboes ≤3.1 + ES=0 or 1 (excluding friability) |
| Histologic – Endoscopic Mucosal Remission (HEMR) | Geboes ≤2B.0 + ES=0 or 1 (excluding friability) |
| Bowel Urgency Clinically Meaningful Improvement (CMI) | Change from baseline in UNRS≥3 in patients with UNRS≥3 at induction baseline |
| Bowel Urgency Numeric Rating Scale (UNRS) | The bowel UNRS is a patient-reported single item that measures the severity of bowel urgency, which is the sudden or immediate need to have a bowel movement, over the past 24 hours, using an 11-point scale ranging from 0 (“no urgency”) to 10 (“worst possible urgency”). Patients were provided an electronic diary tool during screening to record information on the severity of bowel urgency on a daily basis. Weekly scores for each patient were calculated by averaging available daily entries over a 7-day period and rounding to the nearest integer. If fewer than 4 days of data were available, then the patient’s data was considered missing for that week. |
| Bowel Urgency Remission | UNRS=0 or 1, |
| Bowel Urgency Change from Baseline | Change from baseline in Urgency Numeric Rating Scale score; range 0 to 10; a lower score indicates less severe bowel urgency |

**Supplementary Table 2: Clinical Response and Remission Rates at Weeks 24 and 52 for Patients of the Bio-Failed and Not Bio-Failed Population Receiving Mirikizumab Extended Induction**

| **Outcome** | **W24 Extended Induction**  **Bio-failed Responders**  **N=147** | **W24 Extended Induction**  **Not Bio-failed Responders**  **N=125** |
| --- | --- | --- |
| Clinical Response, n (%) | 68 (46.3) | 78 (62.4) |
| Clinical Remission, n (%) | 12 (8.2) | 19 (15.2) |
| **Outcome** | **W52 Extended Induction**  **Bio-failed Responders**  **N=66** | **W52 Extended Induction**  **Not Bio-failed Responders**  **N=78** |
| Clinical Response, n (%) | 46 (69.7) | 58 (74.4) |
| Clinical Remission, n (%) | 23 (34.8) | 29 (37.2) |

***Supplementary Table 3: Corticosteroid-free Remission, Extended Induction Corticosteroid-free Clinical Remission, Extended Induction Clinical Response, and Extended Induction Clinical Remission at Week 52***

| **Corticosteroid-free Remission at W52^a^**  n (%) | | | **Extended Induction Corticosteroid-free Clinical Remission at W52^b^**  n (%) | | |
| --- | --- | --- | --- | --- | --- |
| *All*  *N=144* | *Bio-failed*  *N=66* | *Not Bio-failed*  *N=78* | *All*  *N=144* | *Bio-failed*  *N=66* | *Not Bio-failed*  *N=78* |
| 43 (29.9%) | 17 (25.8%) | 26 (33.3%) | 51 (35.4) | 22 (33.3%) | 29 (37.2%) |
|  | | | | | |
| **Extended Induction Clinical Response at W52**  n (%) | | | **Extended Induction Clinical Remission at W52**  n (%) | | |
| *All*  *N=144* | *Bio-failed*  *N=66* | *Not Bio-failed*  *N=78* | *All*  *N=144* | *Bio-failed*  *N=66* | *Not Bio-failed*  *N=78* |
| 104 (72.2) | 46 (69.7%) | 58 (74.4%) | 52 (36.1) | 23 (34.8%) | 29 (37.2%) |

| ^a^Corticosteroid-free Remission: Clinical remission at LUCENT-3 W52, achieving symptomatic remission by week 40, and with no corticosteroid use for ≥12 weeks prior to W52 |
| --- |
| ^b^Extended Induction Corticosteroid-free Clinical Remission: Corticosteroid- free remission at LUCENT-3 W52 for at least 90 days among extended induction responder patients who achieved clinical remission. |

| **Supplementary Table 4: Univariable Analysis of Clinical Response using Baseline Demographics and Baseline Disease Characteristics as Predictors at Week 24** | | | | |
| --- | --- | --- | --- | --- |
| **Modified ITT Population- Mirikizumab Induction Non-Responders:**  **Open Label Extended Induction Period** | | | | |
| **Parameter** | **W24**  **Non-Responders (N=126)** | **W24**  **Delayed Responders (N=146)** | **Odds Ratio**  **(LCL, UCL)*^a^*** | **P-value*^b^*** |
| Age, Mean (SD) | 43.2 (14.8) | 44.6 (13.7) | 1.007 (0.990,1.024) | 0.990 |
| Age Group 1, n (%) |  | | | |
| <65  ≥65 | 109 (86.5)  17 (13.5) | 132 (90.4)  14 (9.6) | 0.685 (0.323, 1.453) | 0.313 |
| Age Group 2, n (%) |  | | | |
| <40  ≥40 | 62 (49.2)  64 (50.8) | 57 (39)  89 (61) | 1.508 (0.931,2.442) | 0.091 |
| Sex, n (%) |  | | | |
| Male  Female | 92 (73)  34 (27) | 90 (61.6)  56 (38.4) | 1.673 (0.999, 2.801) | 0.046 |
| Baseline BMI Group, n (%) |  | | | |
| Normal (≥18.5 and <25 kg/m^2)  Underweight (<18.5 kg/m^2)  Overweight (≥25 and <30 kg/m^2)  Obese (≥30 and <40 kg/m^2)  Extreme obese (≥40 kg/m^2) | 64 (50.8)  10 (7.9)  35 (27.8)  16 (12.7)  1 (0.8) | 70 (47.9)  6 (4.1)  45 (30.8)  24 (16.4)  1 (0.7) | 0.569 (0.197,1.648)^c^  1.172 (0.672,2.046)^c^  1.357 (0.662,2.779)^c^  0.915 (0.056,14.933)^c^ | 0.615 |
| Tobacco, n (%) |  | | | |
| Current  Former  Never | 5 (4)  34 (27)  87 (69) | 4 (2.7)  45 (30.8)  97 (66.4) | 1.603 (0.401,6.413)^c^  1.355 (0.353,5.198)^c^ | 0.698 |
| Disease Duration, n (%) |  | | | |
| <1 years  ≥1 to <3 years  ≥3 to <7 years  ≥7 years | 10 (7.9)  30 (23.8)  35 (27.8)  51 (40.5) | 9 (6.2)  38 (26)  34 (23.3)  65 (44.5) | 1.393 (0.503, 3.863)^c^  1.073 (0.388, 2.966)^c^  1.404 (0.531, 3.712)^3^ | 0.745 |
| Disease Location, n (%) |  | | | |
| Pancolitis  Proctitis/Left-side colitis | 62 (49.2)  64 (50.8) | 54 (37)  92 (63) | 1.644 (1.013, 2.669) | 0.042 |
| Disease Duration, Mean (SD) | 7.41 (6.64) | 7.798 (6.947) | 1.008 (0.974, 1.045) | 0.974 |
| Prior Biologic or Tofacitinib Failure, n (%) |  | | | |
| ≥2  1  0 | 46 (36.5)  33 (26.2)  47 (37.3) | 45 (30.8)  23 (15.8)  78 (53.4) | 0.717 (0.366,1.405)^c^  1.688 (0.976,2.918)^c^ | 0.017 |
| Prior Biologic or Tofacitinib Group, n (%) |  | | | |
| Failed  Not failed | 79 (62.7)  47 (37.3) | 68 (46.6)  78 (53.4) | 1.917 (1.179, 3.115) | 0.008 |
| Prior Anti-TNF Failure Group |  | | | |
| Failed  Not failed | 70 (55.6)  56 (44.4) | 65 (44.5)  81 (55.5) | 1.552 (0.961, 2.507) | 0.069 |
| Prior Vedolizumab Group |  | | | |
| Failed  Not failed | 44 (34.9)  82 (65.1) | 37 (25.3)  109 (74.7) | 1.574 (0.933, 2.655) | 0.085 |
| Prior Tofacitinib Group |  | | | |
| Failed  Not failed | 10 (7.9)  116 (92.1) | 8 (5.5)  138 (94.5) | 1.466 (0.56, 3.833) | 0.417 |
| Baseline Use of Oral Aminosalicylates |  | | | |
| No  Yes | 37 (29.4)  89 (70.6) | 33 (22.6)  113 (77.4) | 1.419 (0.823, 2.448) | 0.204 |
| Baseline immunomodulator use |  | | | |
| No  Yes | 84 (66.7)  42 (33.3) | 111 (76)  35 (24) | 0.633 (0.373, 1.077) | 0.088 |
| Baseline corticosteroid use |  | | | |
| No  Yes | 73 (57.9)  53 (42.1) | 81 (55.5)  65 (44.5) | 1.104 (0.682, 1.786) | 0.684 |
| Baseline Modified Mayo Score Group |  | | | |
| [<6]  [7-9] | 55 (43.7)  71 (56.3) | 63 (43.2)  83 (56.8) | 0.881 (0.516, 1.504) | 0.934 |
| Baseline Endoscopic Score |  | | | |
| 2  3 | 33 (26.2)  93 (73.8) | 42 (28.8)  104 (71.2) | 0.881 (0.516, 1.504) | 0.635 |
| Baseline Stool Frequency Score |  | | | |
| <3  3 | 45 (35.7)  81 (64.3) | 58 (39.7)  88 (60.3) | 0.845 (0.516, 1.383) | 0.496 |
| Baseline Rectal Bleeding Score |  | | | |
| <2  ≥2 | 71 (56.3)  55 (43.7) | 70 (47.9)  76 (52.1) | 1.397 (0.866, 2.256) | 0.166 |
| Baseline Faecal Calprotectin Group |  | | | |
| >250 ug/g  ≤250 ug/g | 104 (82.5)  3 (2.4) | 113 (77.4)  12 (8.2) | 3.276 (0.937,11.453) | 0.030 |
| Baseline C-Reactive Protein Group |  | | | |
| >6 mg/L  ≤6 mg/L | 68 (54)  57 (45.2) | 61 (41.8)  83 (56.8) | 1.617 (0.998, 2.62) | 0.049 |
| Baseline Faecal Calprotectin, Median (Q1, Q3) | 1658 (934, 2911) | 1458 (514, 2905) | 0.848 (0.686, 1.034) | 0.686 |
| Baseline CRP, Median (Q1, Q3) | 7.2 (3.1, 15.4) | 4.25 (1.7, 10.4) | 0.699 (0.541, 0.887) | 0.541 |
| Baseline IBDQ Total Score, Mean (SD) | 131.08 (33.16) | 131.444 (33.848) | 1.000 (0.993,1.008) | 0.993 |
| Bowel Urgency NRS, Mean (SD) | 6.167 (2.258) | 6.233 (2.143) | 1.014 (0.909,1.131) | 0.909 |

*^a^*Odds ratio indicates quantum of change in response variable due to change of level (categorical) or one unit (continuous) in predictor variable.

*^b^*P-value determined from Likelihood Ratio Test for full model.

*^c^*Odds ratios calculated for each level of response compared to reference category

Abbreviations: BMI=body mass index; IBDQ=Inflammatory Bowel Disease Questionnaire; LCL= Lower Confidence Limit; N/n=number patients; Q=quartile; SD=standard deviation; TNF=Tumor Necrosis Factor; UCL= Upper Confidence Limit; UNRS=Urgency Numeric Rating Scale.

Odds ratios and p-values based on logistic regression with Firth correction. A log+1 transform was applied to C-reactive protein & faecal calprotectin data prior to regression due to skewed profile of data. Proctitis included in “left-side colitis” category.

**Supplementary Table 5: Univariable Analysis of Clinical Response using LUCENT-1 Week 12 Disease Activity as Predictors at Week 24**

| **Modified ITT Population- Mirikizumab Induction Non-Responders:**  **Open Label Extended Induction period** | | | | |
| --- | --- | --- | --- | --- |
| **Parameter** | **W24**  **Extended Induction**  **Non-Responders (N=126)** | **W24**  **Extended Induction**  **Responders (N=146)** | **Odds Ratio (LCL, UCL)*^a^*** | **P-value** |
| Modified Mayo Score |  | | | |
| [0-3]  [4-6]  [7-9] | 7 (5.6)  73 (57.9)  44 (34.9) | 11 (7.5)  97 (66.4)  37 (25.3) | 0.868 (0.322, 2.342)^b^  0.552 (0.195, 1.563)^b^ | 0.196 |
| Mayo Endoscopic Score, Mean (SD) | 2.696 (0.496) | 2.428 (0.734) | 0.507 (0.328, 0.745) | 0.001 |
| Mayo Endoscopic Score (Categorical) |  | | | |
| 3  2  0,1 | 89(70.6)  34(27)  2(1.6) | 82(56.2)  44(30.1)  19(13) | 1.399 (0.816, 2.398)^b^  8.467 (2.128, 33.686)^b^ | 0.0003 |
| Bowel Urgency NRS, Mean (SD) | 5.317 (2.389) | 4.76 (2.425) | 0.909 (0.820, 1.003) | 0.057 |
| IBDQ Total Score, Mean (SD) | 148.81 (35.195) | 159.179 (32.23) | 1.009 (1.002, 1.017) | 0.012 |
| Bowel Urgency NRS Improvement from Baseline |  | | | |
| No  Yes | 100 (79.4)  26 (20.6) | 110 (75.3)  36 (24.7) | 1.252(0.706,2.219) | 0.4296 |
| Bowel Urgency Remission |  | | | |
| No  Yes | 119 (94.4)  7 (5.6) | 130 (89)  16 (11) | 2.006 (0.803, 5.011) | 0.1053 |
| Faecal calprotectin, Median (Q1,Q3) | 1415 (505, 2327.5) | 659 (274, 1966) | 0.739 (0.609, 0.878) | 0.001 |
| Faecal calprotectin Group |  | | | |
| >250 ug/g  ≤250 ug/g | 111 (88.1)  12 (9.5) | 109 (74.7)  32 (21.9) | 2.644 (1.299, 5.38) | 0.004 |
| C-Reactive Protein, Median (Q1,Q3) | 4.51 (1.9,7.8) | 2.64 (1.0, 6.6) | 0.694 (0.519, 0.910) | 0.008 |
| C-Reactive Protein Group |  | | | |
| ≤6 mg/L  >6 mg/L | 81 (64.3)  44 (34.9) | 106 (72.6)  39 (26.7) | 0.68 (0.404, 1.142) | 0.141 |
| Symptomatic Remission |  | | | |
| No  Yes | 118 (93.7)  8 (6.3) | 141 (96.6)  5 (3.4) | 0.545 (0.175, 1.703) | 0.260 |
| Endoscopic Remission |  | | | |
| No  Yes | 124(98.4)  2(1.6) | 127(87)  19(13) | 7.621 (1.936, 30.005) | <0.001 |
| Histologic Improvement |  | | | |
| No  Yes | 119 (94.4)  7 (5.6) | 113 (77.4)  33 (22.6) | 4.704 (2.027, 10.917) | <0.001 |
| Symptomatic Response |  | | | |
| No  Yes | 95 (75.4)  31 (24.6) | 94 (64.4)  52 (35.6) | 1.683 (0.993, 2.853) | 0.0482 |
| Histologic-Endoscopic Mucosal Improvement (HEMI) |  | | | |
| No  Yes | 126 (100)  0 (0) | 133 (91.1)  13 (8.9) | 25.623 (1.353, 485.346) | 0.0001 |
| Histologic-Endoscopic Mucosal Remission (HEMR) |  | | | |
| No  Yes | 126 (100)  0 (0) | 136 (93.2)  10 (6.8) | 19.516 (0.982, 387.745) | 0.0006 |

*^a^*Odds Ratio indicates quantum of change in response variable due to change of level (categorical) or one unit (continuous) in predictor variable.

*^b^*Odds Ratios calculated for each level of response compared to reference category.

Abbreviations: IBDQ=Inflammatory Bowel Disease Questionnaire; LCL= lower confidence limit; N/n=number patients; Q=quartile; SD=standard deviation; TNF=Tumor Necrosis Factor; UCL=upper confidence limit; UNRS=Urgency Numeric Rating Scale.

Odds Ratios and p-values based on univariable logistic regression with Firth correction. A log+1 transform was applied to CRP and fCal data prior to regression due to skewed profile of data.

**Supplementary Table 6: Univariable Analysis of Clinical Response using Change in LUCENT-1 Week 12 Disease Activity as Predictors at Week 24**

| **Modified ITT Population- Mirikizumab Induction Non-Responders:**  **Open Label Extended Induction period** | | | | |
| --- | --- | --- | --- | --- |
| **Parameter** | **W24**  **Extended Induction**  **Non-Responders (N=126)** | **W24**  **Extended Induction**  **Responders**  **(N=146)** | **Odds Ratio**  **(LCL, UCL)*^a^*** | **P-value** |
| Faecal Calprotectin (Change), Median (Q1, Q3) | -340 (-1377, 493.5) | -532 (-1431.2, 177.5) | 1.082  (0.477, 2.582) | 0.824 |
| C-Reactive Protein (Change), Median (Q1, Q3) | -2.505 (-7.3, 0.2) | - 0.71 (-5.1, 0.4) | 6.780  (0.424, 154.823) | 0.172 |
| IBDQ Total Score (Change), Mean (SD) | 17.536 (30.066) | 27.532 (29.514) | 1.011  (1.003, 1.020) | 0.006 |
| Urgency NRS(Improvement), Mean (SD) | 0.849 (2.329) | 1.473 (1.994) | 1.143  (1.024, 1.290) | 0.017 |
| Modified Mayo Score (Improvement), Mean (SD) | 0.484 (1.172) | 1.034 (1.023) | 1.599  (1.274, 2.080) | <0.001 |
| Mayo Endoscopic Score (Improvement), Mean (SD) | 0.04 (0.514) | 0.29 (0.6) | 2.245  (1.450, 3.734) | <0.001 |

*^a^*Odds ratio indicates quantum of change in response variable due to change of level (categorical) or 1 unit (continuous) in predictor variable.

Abbreviations: IBDQ=Inflammatory Bowel Disease Questionnaire; LCL= lower confidence limit; N=number patients; Q=quartile; SD=standard deviation; UCL=upper confidence limit; Urgency NRS=Urgency Numeric Rating Scale.

Odds ratios and p-values based on univariable logistic regression with Firth correction. A log(x+1-min(x)) transform was applied to CRP and fCal data prior to regression due to skewed profile of data.

**Supplementary Table 7: Multivariable Analysis of Clinical Response using Baseline Demographics, Disease Characteristics and LUCENT-1 Week 12 Disease Activity and Improvement as Predictors of Clinical Response at Week 24**

| **Modified ITT Population- Mirikizumab Induction Non-Responders:**  **Open Label Extended Induction period** | | | | | |
| --- | --- | --- | --- | --- | --- |
| **Parameter** | **W24 Non-Responders (N=126)** | **W24 Delayed Responders (N=146)** | **Coefficient** | **Odds Ratio**  **(LCL, UCL)^a,b^** | **P-value ^b^** |
| Intercept |  |  | -1.879 |  |  |
| Week 12 Modified Mayo Score (Improvement), Mean (SD) | 0.484 (1.172) | 1.034 (1.023) | 0.411 | 1.509 (1.108, 2.055) | 0.009 |
| Week 12 Histologic Improvement |  | | | | |
| No  Yes | 119 (94.4)  7 (5.6) | 113 (77.4)  33 (22.6) | 1.367 | 3.922  (1.441, 10.671) | 0.007 |
| Age Group |  | | | | |
| <40  ≥40 | 62 (49.2)  64 (50.8) | 57 (39)  89 (61) | 0.804 | 2.235  (1.197, 4.176) | 0.012 |
| Week 12 Faecal Calprotectin Group |  | | | | |
| >250  ≤250 | 111 (88.1)  12 (9.5) | 109 (74.7)  32 (21.9) | 0.95 | 2.587  (1.015, 6.593) | 0.046 |
| Prior Biologic or Tofacitinib Failure |  | | | | |
| ≥2  1  0 | 46 (36.5)  33 (26.2)  47 (37.3) | 45 (30.8)  23 (15.8)  78 (53.4) | 0.137 | 0.462 (0.193, 1.107)  1.147 (0.57, 2.308) | 0.017 |
| Baseline immunomodulator use |  | | | | |
| No  Yes | 84 (66.7)  42 (33.3) | 111 (76)  35 (24) | 0.688 | 1.989 1.002, 3.95) | 0.049 |
| Week 12 IBDQ Total Score (Improvement), Mean (SD) | 17.536 (30.066) | 27.532 (29.514) | 0.012 | 1.012 (1.001, 1.023) | 0.035 |
| Baseline C-Reactive Protein Group |  | | | | |
| >6 mg/L  ≤6 mg/L | 68 (54)  57 (45.2) | 61 (41.8)  83 (56.8) | 0.497 | 1.643 (0.891, 3.031) | 0.112 |

*^a^*Odds ratio indicates quantum of change in response variable due to change of level (categorical) or one unit (continuous) in predictor variable.

**^b^** Odds ratios and p-values based on stepwise multivariable logistic regression with Firth correction. For the stepwise regression, CRP and fCal continuous variables were omitted in favor of corresponding categorical variables. HEMI and HEMR were omitted due to sparse data. The week 12 Mayo Endoscopic categorical variable was omitted as corresponding continuous variable was included. Week 12 Mayo Modified Score and Baseline Endoscopic Score were omitted due to collinearity with week 12 endoscopic remission.

Abbreviations: BMI=body mass index; IBDQ=Inflammatory Bowel Disease Questionnaire; IV=intravenous; N=number patients; Q=quartile; SC=subcutaneous; SD=standard deviation; TNF=Tumor Necrosis Factor; UC=ulcerative colitis; UNRS=Urgency Numeric Rating Scale.

**Supplementary Table 8: Safety Overview for Mirikizumab Treated Patients For Initial Induction, Extended Induction and Maintenance**

| **Safety Overview^a^** | | | | |
| --- | --- | --- | --- | --- |
| **​** | **Initial Induction​**  **Mirikizumab**  **300 mg IV​** | **Extended Induction​**  **Mirikizumab**  **300 mg IV​** | **Maintenance​**  **Mirikizumab 200 mg SC​** | |
|  | **All Mirikizumab Patients**​  **(N=958)**​ | **Mirikizumab**  **Non-responders at Week 12**​  **(N=313)**​ | **Mirikizumab Induction Responders**​**at Week 12**​  **(N=389)**​ | **Mirikizumab Extended Induction Responders^b^**​  **(N=171)**​ |
| **TEAE, n (%)​** | 426 (44.5)​ | 120 (38.3)​ | 251 (64.5)​ | 99 (57.9) ​ |
| **TEAE by severity, n (%)​**  **Mild​**  **Moderate ​**  **Severe​** | ​  262 (27.3)​  143 (14.9)​  21 (2.2)​ | ​  67 (21.4)​  43 (13.7)​  10 (3.2)​ | ​  148 (38.0)​  87 (22.4)​  16 (4.1)​ | ​  61 (35.7) ​  34 (19.9) ​  4 (2.3) ​ |
| **SAE, n (%)​** | 27 (2.8)​ | 17 (5.4)​ | 13 (3.3)​ | 6 (3.5) ​ |
| **Discontinuation due to AE, n (%)​** | 15 (1.6)​ | 10 (3.2)​ | 6 (1.5)​ | 4 (2.3) ​ |
| **Deaths, n (%)​** | 0​ | 0​ | 0​ | 0​ |

^a^ Data are presented for the safety population, defined as all randomized/assigned patients who received ≥1 dose of study drug; some of these patients were excluded from the efficacy mITT population because of an eCOA transcription error; ^b^ Delayed responders are non-responders to initial induction therapy at week 12 who responded after 12 weeks of extended induction therapy at week 24**​**

Abbreviations: IV=Intravenous, n/N= number of patients, TEAE= Treatment emergent adverse event, AE=adverse event
